# Work-related stress and psychosomatic medicine

**DOI:** 10.1186/1751-0759-4-4

**Published:** 2010-05-26

**Authors:** Mutsuhiro Nakao

**Affiliations:** 1 Department of Hygiene and Public Health, Teikyo University School of Medicine, Tokyo, Japan; 2 Division of Psychosomatic Medicine, Teikyo University Hospital, Tokyo, Japan

## Abstract

This article introduces key concepts of work-related stress relevant to the clinical and research fields of psychosomatic medicine. Stress is a term used to describe the body's physiological and/or psychological reaction to circumstances that require behavioral adjustment. According to the Japanese National Survey of Health, the most frequent stressors are work-related problems, followed by health-related and then financial problems. Conceptually, work-related stress includes a variety of conditions, such as overwork, unemployment or job insecurity, and lack of work-family balance. Job stress has been linked to a range of adverse physical and mental health outcomes, such as cardiovascular disease, insomnia, depression, and anxiety. Stressful working conditions can also impact employee well-being indirectly by directly contributing to negative health behaviors or by limiting an individual's ability to make positive changes to lifestyle behaviors, such as smoking and sedentary behavior. Over the past two decades, two major job stress models have dominated the occupational health literature: the job demand-control-support model and the effort-reward imbalance model. In both models, standardized questionnaires have been developed and frequently used to assess job stress. Unemployment has also been reported to be associated with increased mortality and morbidity, such as by cardiovascular disease, stroke, and suicide. During the past two decades, a trend toward more flexible labor markets has emerged in the private and public sectors of developed countries, and temporary employment arrangements have increased. Temporary workers often complain that they are more productive but receive less compensation than permanent workers. A significant body of research reveals that temporary workers have reported chronic work-related stress for years. The Japanese government has urged all employers to implement four approaches to comprehensive mind/body health care for stress management in the workplace: focusing on individuals, utilizing supervisory lines, enlisting company health care staff, and referring to medical resources outside the company. Good communications between occupational health practitioners and physicians in charge in hospitals/clinics help employees with psychosomatic distress to return to work, and it is critical for psychosomatic practitioners and researchers to understand the basic ideas of work-related stress from the viewpoint of occupational health.

## Introduction

Stress is a term used to define the body's physiological and/or psychological reaction to circumstances that require behavioral adjustment. According to the Japanese National Survey of Health in 2004 [1], 49% of individuals aged 12 years or older reported experiencing stress in their daily lives. This survey examined stress in 28 domains, including work, family, and neighborhood relationships, as well as living-, social-, financial-, and health-related situations. Work-related problems were the most frequent stressors, followed by health-related and then financial problems.

The Japanese Society of Psychosomatic Medicine defines "psychosomatic illness" as any physical condition with organic or functional damage affected by psychosocial factors in the process of its onset or development [2]. This definition largely corresponds to that given in the most recent version of the Diagnostic and Statistical Manual of Mental Disorders, Fourth Edition, Text Revision (DSM-IV-TR), published by the American Psychiatric Association [3]: "psychosocial factors affecting general medical conditions (code 316.00)." In the previous edition of the DSM (DSM-III) and its revision (DSM-III-R) [4], the psychosocial stressor content was defined according to severity, as shown in Table 1. In our previous study, which used axis IV of the DSM-III-R in a psychosomatic outpatient clinic (n = 868) [5], the majority of patients had mild psychosocial stressors (58%), followed by moderate (21%), none (10%), and severe stressors (5%). In the DSM-IV, axis IV has changed from rating the severity of psychosocial stressors to a simple categorization of psychosocial stressors, as shown in Table 2. Evaluating patients according to this new axis IV (n = 564) showed that the most frequent psychosocial stressors were occupational problems (23%), followed by issues related to the primary support group (21%), social environment (5%), and education (5%) [5].

**Table 1:** Severity of psychosocial stressors in adults: DSM-III-R axis IV [4].

	Examples of psychosocial stressors in adulthood	
	
Severity	Acute events	Enduring circumstances
None	None	None

Mild	Broke up with boyfriend/girlfriend	Family arguments
	Started or graduated from school	Job dissatisfaction
	Child left home	Residence in high-crime region

Moderate	Marriage	Marital discord
	Marital separation	Serious financial problems
	Loss of job	Trouble with boss
	Miscarriage	Being a single parent

Severe	Divorce	Unemployment
	Birth of first child	Poverty

Extreme	Death of spouse	Serious chronic illness
	Serious physical illness diagnosed	Ongoing physical or sexual abuse
	Victim of rape	

Catastrophic	Death of child	Captivity as hostage
	Suicide of spouse	Concentration camp experience
	Devastating natural disaster	

**Table 2:** Categories of psychosocial and environmental problems: DSM-IV-TR axis IV [3.]

Category
Problems with primary support group
Problems related to the social environment
Educational problems
Occupational problems (examples below)
Unemployment
Threat of job loss
Stressful work schedule
Difficult working conditions
Job dissatisfaction
Job change
Discord with boss or co-workers
Housing problems
Economic problems
Problems with access to health care services
Problems related to interaction with the legal system/crime
Other psychosocial and environmental problems

Although psychosomatic patients frequently identify work-related problems, these stressors have typically been considered to be relatively mild in severity. For example, acute events, such as job loss or retirement, were regarded as moderate psychosocial stressors in the DSM-III-R assessment, whereas familial events, such as divorce or birth of a first child, were regarded as severe stressors. One of the reasons for such rating criteria is that, while work-related stress is common, it is difficult to assess diagnostically. However, the working environment in Japan and other countries has been changing dramatically. Many employees have been forced to work harder because of ongoing business restructuring and some have suffered from psychosomatic symptoms caused by their work, while others have committed suicide and have been officially acknowledged as victims of depression caused by overwork. Thus, psychosomatic clinicians should pay particular attention to work-related stress when evaluating patients.

Work-related stress includes the concepts of job stress, employment status, job insecurity, and lack of work-family balance. This article introduces key work-related stress concepts (i.e., the job stress model and effects of unstable job conditions on health) relevant to the clinical and research fields of psychosomatic medicine.

### Concept of job stress

Job stress is a substantial and growing concern for workers, their advocates, employers, occupational health and safety regulators, and workers' compensation programs [6,7]. The US National Institute for Occupational Safety and Health defines job stress as "the harmful physical and emotional responses that occur when the requirements of a job do not match the capabilities, resources, or needs of the worker" [8]. Job stress has been linked to a range of adverse physical and mental health outcomes, including cardiovascular disease [9,10], insomnia [11], depression, and anxiety [12]. Stressful working conditions can also impact employee well-being by directly contributing to negative health behaviors or by indirectly limiting an individual's ability to make positive changes to lifestyle behaviors, such as smoking and sedentary behavior [13].

Job stress can result from the job itself (e.g., heavy workload, low input into decision making) or the social and organizational contexts in which the job is performed (e.g., poor communication, interpersonal conflict). There is considerable variation in the way workers perceive and respond to the environments in which they work. Personal (e.g., coping skills) and situational variables (e.g., support from supervisors) influence the onset and duration of job stress, and circumstances that one person finds demanding and stressful may be perceived by others as challenging and simulating [14].

**Figure 1:**
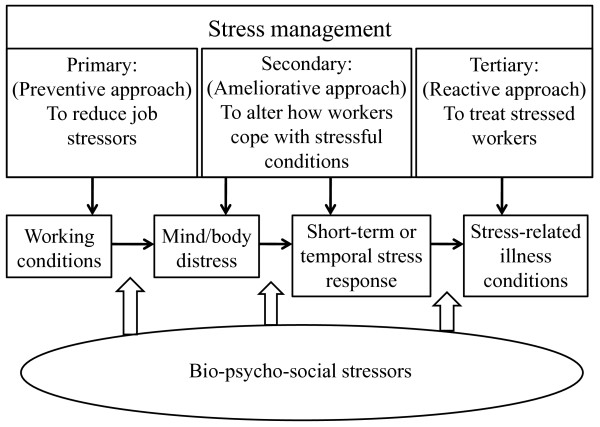
**Job stress process and systemic approach to stress management**. This figure was adapted from models that were previously proposed [6,15,16].

Figure 1 shows the process underlying development of job stress as well as a systemic approach to reducing job stress [6,15,16] . Workplace stressors can be addressed through occupational health and safety: stress can arise through a combination of work- and non-work-related circumstances and can be addressed by integrating occupational health and safety, health promotion, and other approaches, including psychosomatic medicine. Short-term and temporary responses can be physiological (e.g., elevated blood pressure), psychological (e.g., depression), or behavioral (e.g., excessive alcohol drinking). Over the long-term, such responses can lead to disease conditions of a physical (e.g., hypertension), psychological (e.g., depressive disorder), or behavioral nature (e.g., alcoholism).

### Job stress model

Over the past two decades, two major job stress models have dominated the occupational health literature: the job demand-control-support model proposed by Karasek and Theorell [17] and the effort-reward imbalance (ERI) model developed by Siegrist [18].

**Figure 2:**
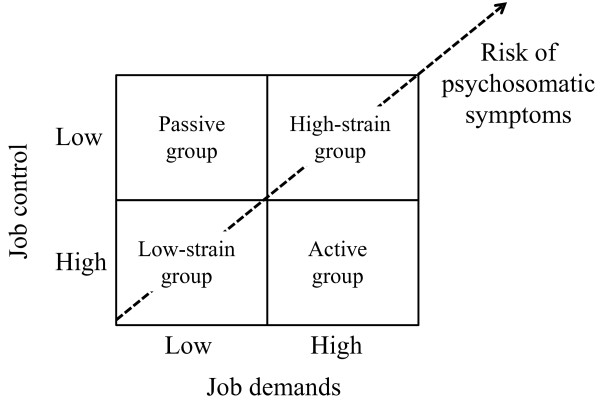
**Job strain groups according to job demands and control**. This figure was adapted from the models that were previously proposed [19,20].

Karasek described two dimensions of job demand and control. Job control or decision latitude comprises decision authority (controllability over work) and skill discretion (variety of work and opportunity for use of skills). The ratio of job demand to job control is often called job strain, and Figure 2 shows four categories of jobs, divided according to job strain (i.e., high-strain, active, low-strain, and passive) [19,20]. The high-strain group is at the highest risk: psychosomatic symptoms are predicted to be severe when the psychological demands of a job are high and the workers' decision latitude is low because the worker lacks the resources to deal with demands. The active group may experience intensely demanding jobs, but the workers have sufficient control over their activities and the freedom to use available skills. Thus, average psychological strain and active leisure time are predicted. The low-strain group, experiencing few psychological demands and high levels of control, should have below-average levels of psychological strain and lower risk of ill health because these individuals have relatively few challenges, and decision latitude allows them to respond optimally to these challenges. The passive group, characterized by low demands and low control, is predicted to be demotivated, which may induce atrophy of skills and abilities, but only average levels of psychological strain and health risk are expected [21].

Further work by Johnson and Thorell [17,22] added the important dimension of occupational social support to Karasek's model, as it had been noted that support from supervisors and co-workers buffered the effects of high demands and low control. This integrated model is called the demand-control-support model. In this context, determining whether a social network provides support to mediate psychosocial strain is decisive in the development of illness [23]. Working conditions that include both high strain and low social support (i.e., iso-strain) have the greatest negative impact.

In Siegrist's ERI model, high-cost and low-gain work conditions are particularly stressful. Work offers opportunities to gain self-esteem, efficacy, and integration. According to the social exchange theory, workers invest effort and expect these rewards in return. If there is an imbalance in the expected exchange that prevents workers from receiving rewards, then psychological distress occurs, accompanied by physiological arousal. Thus, one risk factor for ill health is a combination of high effort at work, which may entail intrinsic effort and innate competitiveness and hostility, combined with high extrinsic job demands and little reward in terms of salary, promotion, or esteem.

### Example of job stress study

A standardized questionnaire, such as the Job Content Questionnaire (JCQ), is frequently used as an assessment tool for the job demand-control-support model [24]. The reliability and validity of the Japanese version of the JCQ have been demonstrated [25]. Using this version of the JCQ, we conducted a cross-sectional study of work-related stress and arterial stiffness in 396 male workers, aged 24 to 39 years, who were employed in a Japanese information-service company [26]. Brachial-pulse wave velocity (baPWV) was used to assess the degree of arteriosclerosis, and the Profile of Mood State (POMS) was used to assess mood. Results indicated that the two POMS scales, tension-anxiety and anger-hostility, were significantly higher in the high-demand and high-strain groups compared with the low-demand and low-strain groups (Table 3), suggesting that work-related stress affects mood. However, job strain was negatively associated with baPWV, even after controlling for significant cardiovascular disease (CVD) risk factors, such as age, heart rate, and serum noradrenaline levels, as shown in Table 3. These effects were in direct opposition with what was predicted based on the job strain hypothesis, which suggests that workers with high job strain have an increased risk for CVD [27]. However, the results of the Coronary Artery Risk Disease in Young Adults study [28] were compatible with our study, showing inverse associations with risk factors for high job demands, low control, and job strain in young adults. In our study [26], subjects in the low-job-strain group tended to have higher diastolic blood pressure, body mass index, serum levels of total cholesterol, blood sugar levels, and ethanol consumption compared with the high-job-strain group. Thus, it is possible that subjects in the low-job-strain group coped with the distress of overwork by adopting unhealthy lifestyles. Another possible explanation is that age-related cultural factors might affect the relationship between the JCQ and baPWV. The inconsistency of the current results with other studies raises concerns about applying the JCQ to Japanese workers and suggests that other factors, such as cultural influence and age, should also be considered.

**Table 3:** Effects of job stress, cardiovascular disease risk factors, and mood state on brachial-ankle pulse velocity in 396 male workers.

	Regresion analysis	
	
Independent variables	Univariate	**Multivariate**^**a**^
Job Content Questionnaire^b^		
Job demands	NS^c^	(-)*
Job control	(+)**	(+)**
Social support	NS	-
CVD risk factors		
Age	(+)***	(+)***
Heart	(+)***	(+)***
Body mass index	(+)**	NS
Serum lipid levels		
Total cholestrerol	(+)***	NS
Triglyceride	(+)***	-
Fasting glucose levels	(+)*	NS
Serum catecholamine levels		
Adrenaline	NS	-
Noradrenaline	(+)**	(+)*
Dopamine	NS	-
Cigarettes smoked per day	NS	-
Profile of Mood State		
Tension-Anxiety	NS	-
Anger-Hostility	NS	-

^a ^Variables with *p *< 0.05 in the univariate analysis were selected as independent variables in the multivariate analysis. Serum triglyceride levels were excluded in the multivariate analysis because of the collinearity between total cholesterol and triglyceride.

^b ^The reasons for the inverse relationship between higher brachial-ankle pulse velocity and higher job strain (i.e., higher job demands and lower job control) are discussed in the text.

^c ^NS, not significant (*p *> 0.05).

*p < 0.05, **p < 0.01, ***p < 0.001

This table was completed by reanalyzing data from our previous study [26].

**Figure 3:**
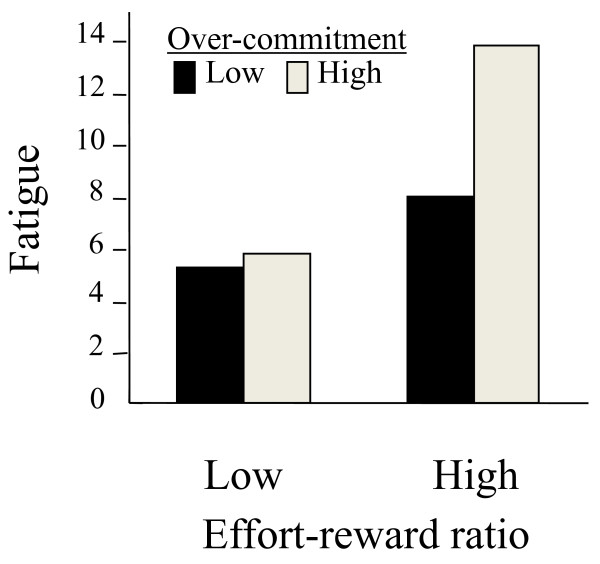
**Interaction of over-commitment and the relationship between fatigue and effort-reward imbalance**. Data from our previous study [31] of 95 male overtime workers were re-analzyed using the DSM-IV-TR. Values one standard deviation above and below the mean were used to represent typical high and low scores.

A standardized ERI questionnaire has been developed and includes three main scales: extrinsic effort, reward, and over-commitment [29]. A score for the effort-reward ratio was obtained by calculating the log-transformed ratio of extrinsic effort to reward as a continuous measure. Over-commitment indicates a state of exhaustive coping that reflects continued, frustrated efforts and negative feelings associated with this state. A Japanese version of the ERI questionnaire was developed by Tsutsumi and colleagues [30]. To confirm the applicability of the ERI model of over-commitment to the assessment of fatigue, we investigated 95 male workers in a Japanese information-technology company using the Japanese versions of the ERI questionnaire and the POMS [31]. All had worked overtime, according to the standards of the Ministry of Health, Labor and Welfare in Japan: they worked at least 100 hours during the preceding 1-month period and/or at least 80 hours per month over the preceding 2- to 6-month period. Results showed that the effort-reward ratio was significantly positively correlated with the POMS fatigue scores, and there was a 3-way interaction among over-commitment scores, POMS fatigue scores, and effort-reward ratio. Specifically, the increase in psychological fatigue that accompanied increased ERI scores was greater in workers with higher over-commitment than in those with lower over-commitment, as shown in Figure 3. These results suggest a need for interventions to reduce worker ERI, over-commitment, and fatigue, to improve productivity, and to limit occupational accidents.

### Unemployment and job insecurity

Evidence has consistently indicated that unemployment is associated with increased mortality and morbidity [32-34]. Due to the labor market structure, previous studies defined work status as a dichotomy (i.e., employed or unemployed). However, during the past two decades, a trend toward more flexible labor markets has emerged in the private and public sectors of developed countries. Employers and policy makers have seen labor-market flexibility as a means of improving worker performance and adaptability in the face of technical change and increasing globalization. As a result, the labor market has increasingly moved toward a core-periphery structure in which the core comprises employees with relatively secure permanent jobs, and the periphery consists of the "buffer work force" with various temporary, unstable, and insecure work arrangements, among which the outermost sector has the highest risk for unemployment [35-37].

The firing of temporary workers is an issue of great concern in Japan. The world financial crisis became publicly known around October 2008 with the fall of Lehman Brothers. The Japanese economy depends greatly on the American economy; it is often said, "When America catches a cold, Japan risks pneumonia." In Japan, temporary workers started losing their jobs as a result of the economic recession. Because a temporary employment system has only recently been initiated in Japan, this rapid increase in firings will have a major impact on workers, those seeking jobs, and their family members [38]. The largest issue is that the Japanese health management system, including accident insurance and medical benefits, does not accommodate the variety of current employment patterns. Of great concern, little medical research has focused on possible risks of mental health problems among temporary workers, who face high levels of job insecurity.

Temporary workers often complain that they are more productive but receive lower compensation than permanent workers [39]. Our own research has found that term-limited workers tend to work more hours and experience symptoms of fatigue more frequently than do permanent workers [40]. A significant body of research [41] reveals that temporary workers have reported chronic work-related stress for years, but the recent sudden increase in job insecurity is beyond expectation; it is akin to an acute trauma or sudden-onset disaster that gives temporary workers a "one-two punch" of both acute and chronic stress.

Practitioners of psychosomatic medicine have been seeing many patients who are temporary workers complaining of psychosomatic distress due to job insecurity; indeed, some have already attempted suicide. This situation is not limited to the Japanese population; it is a problem in all industrial countries. The growing frequency with which temporary workers are being fired threatens to create a situation that may be termed "creeping" mental health problems. Thus, in this author's opinion, we have a responsibility to speak about such issues publicly to determine whether temporary working conditions really affect health status.

### Management of job stress

The Japanese government has urged all employers to implement four approaches to comprehensive mind/body health care: focusing on individuals, utilizing supervisory lines, enlisting company health care staff, and referring to medical resources outside of the company [42]. Concerning the fourth approach, medical resources are not limited to psychosomatic practitioners or other clinics/hospitals; for example, employee assistance programs (EAPs) have attracted a great deal of attention in Japan since 2000 as promising medical resources outside the workplace. Originally, EAPs were employer-sponsored systems developed to restore or improve the functioning of workers whose personal problems were affecting job performance [43]. Newer, more comprehensive EAPs engage in identification, assessment, monitoring, referral, short-term counseling, and follow-up activities with regard to the emotional, financial, legal, family, and substance-abuse concerns of employees. In this sense, comprehensive EAPs are new in Japan and primarily target the mental health care of employees. In our cohort study of 283 male Japanese employees who accessed EAP services [44], total scores on the 17-item Hamilton Depression Scale after the 2-year study period decreased significantly on five items: suicidal thoughts, agitation, psychomotor retardation, guilt, and depressed mood. Specifically, 19 (86%) of the 22 workers with a positive response to the suicidal thoughts item at baseline (i.e., score ≥ 1) reported no suicidal thoughts after the 2-year study period (i.e., score = 0). No significant changes were observed in the control group. These data suggest that introducing an EAP may decrease perceived psychosocial stress in a working population.

## Conclusions

Work-related stress is commonly seen in psychosomatic medicine clinics. The job demand-control-support model and the ERI model are recognized as reliable and useful for assessing job stress. Both unemployment and job insecurity are regarded as risk factors associated with increased mortality and morbidity in a variety of physical and psychological disease conditions. A significant body of research reveals that temporary workers have reported chronic work-related stress for years. To manage stress in the workplace, a combination of individual-focused and organization-focused approaches is the most promising [45], and the following four approaches are recommended for comprehensive mind/body health care in the workplace: focusing on individuals, utilizing supervisory lines, enlisting company health care staff, and referring to medical resources outside the company.

In the occupational health field, medical professionals have many roles, including regular health examinations of employees, health consultation with symptomatic employees, and regular monitoring of the work environment to protect all workers. In addition, metabolic syndrome health examinations and special examinations for employees with excessive work schedules are current concerns in the Japanese workplace. Because physicians specializing in psychosomatic medicine can assess both physical and psychological illness, they are often asked to perform such assessments in the workplace. Medicine should not be limited to disease treatment in a hospital; it is also important to prevent disease. To practice psychosomatic medicine in the hospital requires a trusting relationship between the patient and doctor, and both must be aware of the power of the mind-body connection. Communication is the key factor for developing this relationship. This is also true for the relationship between the employee and occupational health physician and between the occupational health physician and the physician in charge at the hospital, who see the same patient in different settings. For example, good communication helps employees with psychosomatic distress to recover and return to work. For this reason, it is critical for psychosomatic practitioners and researchers to understand the basic ideas of work-related stress from the viewpoint of occupational health.

## Competing interests

The author declare that they have no competing interests.

## Authors' contributions

The author wrote the manuscript and holds final responsibility for the decision to submit the manuscript for publication.
